# Parental income and poor mental well-being among Danish high school students: combining national registry data with survey information

**DOI:** 10.1007/s00787-025-02789-4

**Published:** 2025-06-20

**Authors:** Magnus Elias Tarp, Maja Bramming, Mette Lise Lousdal, Janne Tolstrup

**Affiliations:** 1 Department of Clinical Epidemiology, Aarhus University and Aarhus University Hospital, Olof Palmes Allé 43-45, Aarhus N, 8200 Denmark; 2 National Institute of Public Health, Studiestræde 6, Copenhagen, 1455 Denmark

**Keywords:** Mental health, Well-being, Child and adolescence, Young people, Socioeconomic differences, Income

## Abstract

**Background:**

Poor mental well-being can affect the future of young individuals, especially during adolescence. This study aimed to investigate the association between parental income and poor mental well-being in youth aged 15–19, while also exploring whether this relationship differs by gender and follows a specific pattern.

**Methods:**

We conducted a cross-sectional study using data from The Danish National Youth Study, which included 53,038 high school students aged 15–19. This was combined with information from several national registers. Mental well-being was measured using The Short Warwick-Edinburgh Mental Health Well-being Scale, and poor mental well-being was defined as scoring in the lowest decile. Parental income was categorised into quintiles. We used multivariable logistic regression to analyze the data and restricted cubic spline regression to examine the relationship’s shape.

**Results:**

Compared to the highest parental income group, the adjusted odds ratios of poor mental well-being were 1.04 (95% confidence intervals: 0.94–1.15) for Q4, 1.12 (1.01–1.24) for Q3, 1.20 (1.08–1.33) for Q2, and 1.21 (1.08–1.36) for Q1, with no significant differences between genders. In continuous analysis, we found that higher parental income was associated with less poor mental well-being up to the approximately 75th percentile of income; from this level, higher income was not associated with further change in poor mental well-being.

**Conclusion:**

Lower parental income was associated with higher odds of poor mental well-being during adolescence, with no gender differences. The findings suggest an indication of a threshold where lower income becomes more strongly associated with higher odds of poor mental well-being.

## Introduction

Adolescence is a crucial stage of development. Poor mental well-being during this time can have long-term consequences, including lower physical and emotional well-being, reduced life satisfaction, and learning difficulties, which can hinder success in adulthood [1–3]. Consequently, the mental well-being of young individuals plays a crucial role in shaping their future. Previous research highlights that parental income is associated with various mental health outcomes in offspring. For example, Reiss’s systematic review in 2013 found that 52 out of 55 studies reported an inverse relationship between socioeconomic status (SES) and mental health problems in children and adolescents [4]. Furthermore, low parental income has been linked to worse academic performance, lower self-esteem, and a higher risk of behavioural problems [5].

Much of the existing research on parental income and mental health in youth focuses on clinically diagnosed disorders. However, the World Health Organization (WHO) defines good mental health as more than the absence of mental disorders– it is a state of well-being in which individuals can realize their abilities, work, learn, and contribute to their communities [6]. Recognizing this broader perspective is essential for identifying early signs of mental health issues that may not meet the threshold for a clinical diagnosis. Additionally, much of the research on parental income and mental health in children and adolescents has not used comprehensive national registry data.

We aimed to combine Danish national registry data with survey data to explore the relationship between parental income and general mental well-being among adolescents aged 15–19. Unlike prior studies, we focused on identifying early signs and less severe challenges to mental well-being rather than diagnosed disorders. Additionally, we examined whether the association between parental income and mental well-being differs by gender and whether this relationship plateaus at higher income levels.

## Methods

### Study design and setting

This cross-sectional study used data from the Danish National Youth Study 2014 (DNYS 2014), a nationwide survey designed to assess health and well-being among young individuals in Denmark [7, 8]. The DNYS targeted high school and vocational students aged 15–25 years, focusing on those in secondary education across Denmark. For this study, we included only high school students aged 15–19 who had complete data on mental well-being, parental income, and all relevant covariates. Data from DNYS 2014 were linked to national registers using the unique personal identification number assigned to all individuals living in Denmark. Parental income was assessed in 2013, in the year prior to survey response. Participants with a parental income of zero or less were excluded as these families may rely on unregistered income sources or have expenses covered by other means [9]. Participants gave informed consent before completing the Danish National Youth Cohort survey online and linkage to national registers was approved by the Danish Data Protection Agency (No. 2013-54-0526).

### Study variables and data sources

Mental well-being was assessed using the Short Warwick-Edinburgh Mental Health Well-being Scale (SWEMWBS), which includes seven items measuring positive mental states over the past two weeks, rated on a 5-point Likert scale. Poor mental well-being was defined as having a SWEMWBS score in the lowest decile, with a threshold of 18.59. The median SWEMWBS score for the study population was 23.1. The Danish-translated version used in the study has been validated for psychometric properties [10]. Parental income was obtained from the Danish Income Statistics Register [11] and reflects the total pre-tax income for the year 2013, including income from employment, social benefits, private pensions, investment income, and other non-classified income. For this study, it was categorised into quintiles (Q1–Q5), with Q5 (highest income) serving as the reference group. The variable reflects the combined income of all family members living at the same address as the participant as of December 31, 2013.

Country of origin was obtained using the Danish Civil Registration System [12] and categorized as Danish if at least one parent had both Danish citizenship and was born in Denmark. Living arrangements were determined based on DNYS 2014 and grouped into three categories: Those living with parent(s) in a couple, those living with a single parent, or others (i.e., living alone, with siblings, grandparents, foster families, or at an institution). Parental illness or disability was also assessed using DNYS 2014, where participants reported any serious illness or disability of their parents. Parental educational level was obtained from the Danish Education Registers [13] and defined as the highest completed education level among parents and categorized into three levels: short, medium, and long education. Short education included primary school, upper secondary school, or completed vocational school; medium education included shorter higher education or medium-long higher education; and long education included longer higher education or above.

### Statistical analysis

Descriptive analyses characterized the study population. Logistic regression was used to examine the association between parental income and poor mental well-being, presenting odds ratios (ORs) with 95% confidence intervals (CIs) for the quintile groups. We present unadjusted and adjusted analyses (i.e., to isolate the effect of parental income from other covariates). To address limitations in the data collection from DNYS 2014, we included gender and age in the model, as well as the school each student attended. Gender-stratified analyses were performed, with differences evaluated using likelihood-ratio tests for interaction between gender and parental income. To explore whether the association varied with parental income levels, we used a Poisson regression model with restricted cubic splines (5 knots), modelling parental income as a continuous variable. This allowed flexible examination of non-linear trends in the association, using reference points of 250,000 DKK (approximately 33,500 €) for absolute income and the 10th percentile for relative income.

We conducted sensitivity analyses using alternative cut-points for poor mental well-being: a SWEMWBS score of ≤ 20, using the lowest 20% of scores, and the lowest 5% of scores, respectively.

All data management and analysis were performed using Stata software version 18 (StataCorp. 2023. *Stata Statistical Software: Release 18*. College Station, TX: StataCorp LLC).

## Results

In this study, we excluded 8,823 individuals who were older than 19, 1,918 who had missing information on any variable, and 118 who had a parental income of zero or less. After these exclusions, the final study population included 53,038 high school students aged 15 to 19, see Table 1. Of the 53,038 participants, 19,989 (37.7%) were men and 33,049 (62.3%) were women. The median age was 17.6 years. Poor mental well-being, defined as being in the lowest decile of mental health, was observed in 10.3% of participants. This percentage varied by quintile of parental income, with 13.1% in Q1, 11.7% in Q2, 10.2% in Q3, 9.0% in Q4, and 7.9% in Q5. Participants in Q1 had higher proportions of being an immigrant or descendant, having lower parental education levels, and having a parent with an illness or disability compared to participants in Q5. Additionally, young individuals in Q1 were less likely to live with both parents as compared to those in Q5.

**Table 1:** Characteristics of the study population according to quintiles of parental income

	All (*n* = 53,038)	Low income (Q1)	Low-middle income (Q2)	Middle income (Q3)	High-mid income (Q4)	High income (Q5)
**Men** (n, %)	19,989 (37.7)	3,621 (35.9)	3,750 (35.2)	3,906 (36.4)	4,103 (38.1)	4,609 (42.7)
**Age**, yrs median (5th -95th )	17.6 (16.2–18.8)	17.6 (16.2–18.9)	17.6 (16.1–18.8)	17.6 (16.2–18.8)	17.6 (16.2–18.8)	17.6 (16.1–18.8)
**Immigrant or descendant** (n, %)	4,219 (8.0)	2,063 (20.4)	1,312 (12.3)	455 (4.2)	226 (2.1)	163 (1.5)
**Living with:**						
Parent(s) in couple (n, %)	41,587 (78.4)	4,198 (41.6)	7,896 (74.1)	9,586 (88.3)	9,855 (91.6)	10,052 (93.1)
Single Parent (n, %)	9,531 (18.0)	5,018 (49.7)	2,398 (22.5)	862 (8.0)	695 (6.5)	558 (5.2)
Other (n, %)	1,920 (3.6)	887 (8.7)	362 (3.4)	288 (2.7)	211 (2.0)	182 (1.7)
**Parental educational level**^a^:						
Low ^b^ (n, %)	20,041 (37.8)	5,453 (54.0)	5,438 (51.0)	4,472 (41.7)	2,942 (27.3)	1,737 (16.1)
Medium (bachelor level) ^c^ (n, %)	20,790 (39.2)	3,553 (35.2)	3,772 (35.4)	4,954 (46.1)	5,030 (46.7)	3,481 (32.3)
High (master level or higher) ^d^ (n, %)	12,207 (23.0)	1,087 (10.8)	1,446 (13.6)	1,310 (12.2)	2,789 (25.9)	5,575 (51.7)
**Parental illness or disability** (n, %)	8,541 (16.1)	2,259 (22.4)	2,013 (18.9)	1,641 (15.3)	1,407 (13.1)	1,211 (11.3)
**In poor mental well-being group**^e^ (n, %)	5,472 (10.3)	1,326 (13.1)	1,242 (11.7)	1,091 (10.2)	964 (9.0)	849 (7.9)

^a^ Based on the parent with highest obtained educational level, ^b^ Primary School, Upper Secondary School, or Completed Vocational School, ^c^ Shorter Higher Education or Medium Long Education, ^d^ Longer Higher Education, ^e^ Share of decile with lowest mental well-being

The ORs for poor mental well-being were associated with parental income, see Table 2. The association was attenuated after adjusting for other covariates, where individuals in Q1 had 21% higher odds of experiencing poor mental well-being compared to those in Q5 (1.21, 95% CI: 1.08–1.36). The adjusted ORs for Q2, Q3, and Q4 were 1.20 (1.08–1.33), 1.12 (1.01–1.24), and 1.04 (0.94–1.15), respectively. Stratified analyses by gender showed similar trends for both young women and men, with the likelihood-ratio- test showing no interaction between gender and parental income group (*p* = 0.44).

**Table 2:** Association between parental income and poor mental well-being^a^

	% in lowest mental well-being group	Crude Odds Ratio ^b^	(95% CI)	Adjusted Odds Ratio ^c^	(95% CI)
**Overall** (*n* = 53,038)					
High income (Q5)	7.9	1		1	
High-mid income (Q4)	9.0	1.09	(0.99–1.20)	1.04	(0.94–1.15)
Middle income (Q3)	10.2	1.23	(1.12–1.36)	1.12	(1.01–1.24)
Low-mid income (Q2)	11.7	1.43	(1.30–1.57)	1.20	(1.08–1.33)
Low income (Q1)	13.1	1.62	(1.48–1.78)	1.21	(1.08–1.36)
**Young Women** (*n* = 33,049)					
High income (Q5)	10.7	1		1	
High-mid (Q4)	11.5	1.08	(0.97–1.21)	1.03	(0.91–1.15)
Middle income (Q3)	13.1	1.24	(1.11–1.39)	1.12	(1.00-1.25)
Low-mid income (Q2)	14.6	1.41	(1.27–1.57)	1.18	(1.05–1.33)
Low income (Q1)	16.3	1.58	(1.42–1.77)	1.17	(1.03–1.33)
**Young Men** (*n* = 19,989)					
High income (Q5)	4.1	1		1	
High-mid (Q4)	4.8	1.11	(0.90–1.37)	1.08	(0.87–1.33)
Middle income (Q3)	5.1	1.20	(0.97–1.48)	1.11	(0.89–1.38)
Low-mid income (Q2)	6.3	1.49	(1.21–1.82)	1.26	(1.02–1.58)
Low income (Q1)	7.6	1.78	(1.47–2.18)	1.37	(1.08–1.74)

^a^ Poor mental well-being is defined as being in the lowest decile of mental well-being. ^b^ Crude Odds Ratios adjusted for school, gender, and age. ^c^ Odds Ratios adjusted for school, gender, age, ethnicity, parental illness or disability, parental educational level, and whether living with a single parent, parent(s) in couple, or others

**Fig. 1:**
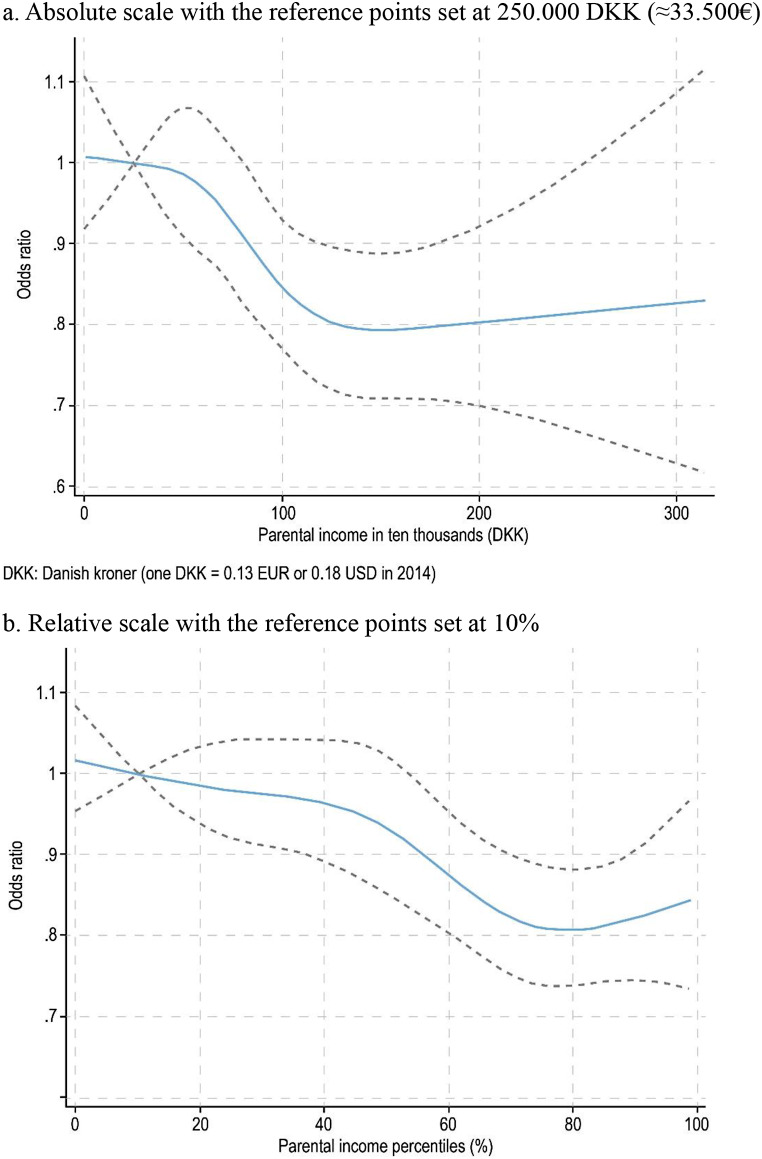
Restricted cubic spline models for predicted odds ratio of poor mental well-being according to parental income. Both fig **a** and **b** are adjusted for age, gender, school, ethnicity, parental illness or disability, parental educational level, living situation (with single parent, parent(s) in a couple, or other). Dashed lines indicate 95% confidence intervals. The reference points used are 250,000 DKK (approximately 33,500 €) for absolute income and the 10th percentile for relative income.

Figure 1 shows the flexible modelling of non-linear trends between parental income and poor mental well-being. On the absolute scale (Fig. 1a), the predicted odds ratio declines slightly from the lowest parental income up to approximately 500,000 DKK (approximately €67,000), after which it decreases more steeply until around 1,500,000 DKK (approximately €200,000). Beyond this threshold, the odds ratio plateaus. On the relative scale (Fig. 1b), the OR decreases slowly from the 1st to the 40th percentile, and then drops more sharply until the 75th percentile.

Sensitivity analyses using alternative cut-points for poor mental well-being showed results consistent with the main findings. Across all definitions, lower parental income was associated with higher odds of poor mental well-being and appeared to have a non-linear pattern showing similar ORs among the high income (Q1) and high-mid groups (Q2) corresponding to the plateau effect for higher income and similar ORs among the low-mid (Q4) and low-income groups (Q5) (Table 3).

**Table 3:** Results of sensitivity analyses changing cut-points for poor mental well-being

	Lowest 10% as cut point*	Lowest 20% as cut point	Lowest 5% as cut point	SWEMWBS score of ≤ 20 as cut point
Overall *n* = 53,038				
High income (Q1)	1	1	1	1
High-mid (Q2)	1.04	1.04	1.10	1.04
Middle income (Q3)	1.12	1.15	1.10	1.15
Low-mid income (Q4)	1.20	1.20	1.22	1.20
Low income (Q5)	1.21	1.19	1.32	1.19

*Cut point used in this paper

## Discussion

This study highlights a significant association between parental income and mental health outcomes among adolescents, revealing that young individuals from lower-income families experience higher odds of poor mental well-being compared to their peers from higher-income backgrounds after adjusting for country of origin, living arrangements, parental educational level, and illness or disability of the parents. Notably, this relationship remains consistent across genders. Furthermore, our findings indicate a nonlinear relationship between parental income and the odds of poor mental well-being in youth. Specifically, we identified a threshold range of parental income between approximately 500,000 DKK (≈ 67,000 €) and 1,500,000 DKK (≈ 200,000 €). Within this range, increases in parental income correlate more strongly with improvements in mental health. This suggests that while financial resources are crucial, their effect may diminish at higher income levels. This hypothesis aligns with theories suggesting that income and mental health is associated only until a certain threshold. According to this view, income influences mental health primarily by enabling people to meet their basic needs [14].

We were unable to find any previous studies that specifically investigated the relationship between parental income and adolescent mental well-being. However, several studies have explored related outcomes such as psychiatric disorders and mental health problems. For instance, a discordant sibling study by Sariaslan et al. [15] concluded that there was no association between parental income and psychiatric disorders. However, the methodology used in their study to address the research question has been questioned [16–19]. Their conclusion not only contrasts with our results but also with previous research, including a systematic review that identified an inverse relationship between socioeconomic status and mental health problems in children and adolescents [4]. This review also indicated that there were no significant gender differences in this relationship, which aligns with our findings. Consistent with our results, both Kinge et al. [20] and Hynek et al. [21] found an inverse relationship between parental income and the risk of mental disorders. Kinge et al. further observed that this relationship was non-linear. However, while Hynek et al. [21] found no gender differences in the association, consistent with our findings, Kinge et al. [20] reported gender specific patterns. This difference may be due to the fact that Kinge et al. explored gender-specific disorders (e.g., ADHD in boys, anxiety and depression in girls), while Hynek et al. and our study explored mental health, in general, which may have obscured gender-specific findings for particular diagnoses.

The main strengths of our study include the large sample size and the ability to link to national registers. This linkage allowed us to gather information on parental income, country of origin, and parental education levels. This use of registry-based avoids the biases that may arise from self-reported information. For instance, parental income was based on documented data, increasing the reliability of our findings.

This study also has limitations. The cross-sectional design prevents us from establishing causal relationships. While we observed an association between parental income and poor mental well-being, the direction of this relationship remains unclear, and reverse causation could be at play. For example, an adolescent with low mental well-being facing difficulties in life may need more care and attention from their parents, which may limit their ability to work. In this way, poor mental well-being in young people could lead to lower parental income, by reducing the parents’ ability to work.

Additionally, while DNYS 2014 had a high participation rate of 85%, the 15% who did not participate could still introduce bias. A study by Pisinger et al. [8] found that participants in DNYS 2014 are more likely to have parents with higher incomes than non-participants. If the non-participants also have lower mental health, as our findings suggest, it could influence the results.

A total of 1,918 individuals were excluded due to missing data on one or more key variables. Previous research by Pisinger and colleagues has shown that non-respondents within the Danish DNYS 2014 were more likely to be male, of non-Danish origin, and have parents with lower incomes and educational levels [8]. This suggests that excluded individuals may have been more socioeconomically disadvantaged, potentially biasing our findings toward a more conservative estimate of the association. Our findings may not fully reflect the mental well-being of all Danish high school students, as they may miss those from more vulnerable backgrounds. Another potential source of bias is the reliance on self-reported data in the DNYS 2014 survey for variables such as living situations, parental illness or disability, and mental well-being. Self-reporting may lead to recall bias or social desirability bias [22, 23]. For instance, respondents might overestimate their mental well-being or underreport parental illness due to stigma. Such biases could result in an underestimation of poor mental well-being in this study.

Additionally, since this study relies on pre-collected data, we were unable to include certain potentially important confounding variables. For instance, Kinge et al. [20] included the number of household members in their analysis, explaining some of the association between parental income and mental disorders. Household size could also influence both parental income and poor mental well-being in young people in this study. Having more siblings has been associated with better well-being [24], while larger family sizes can also lead to lower parental income due to reduced working hours. Since we did not have data on household size, we were unable to account for this potential confounder. If included, it would likely strengthen the observed association between parental income and low mental well-being, resulting in a higher odds ratio. Another approach to account for this would have been to use equivalized disposable income, which accounts for household size [9], but this information was not available. Another thing to notice is that although we refer to our income measure as “parental income” it is technically a measure of total household income and may include income from children and adolescents within the household. We were also unable to identify whether some individuals were siblings. This means we could not account for potential clustering within families, which may violate the assumption of independence in the logistic regression models.

Moreover, examining the role of community resources, such as access to out-of-school activities, mental health services, or supportive school environments, may be essential in mitigating the effects of low parental income on adolescent mental health. These contextual factors could serve as protective buffers, particularly for adolescents from lower-income households, and could be integrated into future studies on the topic.

## Conclusion

This study examined the relationship between parental income and the mental well-being of adolescents aged 15–19, with a focus on identifying early signs and subclinical mental health challenges. The findings show that adolescents from lower-income families have higher odds of experiencing poor mental well-being compared to those from higher-income families. This association does not vary by gender. Additionally, the results suggest a nonlinear relationship between parental income and mental health, with a point where the association levels off or even decreases. By focusing on mental well-being instead of only diagnosed disorders, this study offers valuable insights into early signs of mental health issues in adolescents. These findings may help inform public health interventions and policies in countries with similar welfare systems, supporting efforts to promote the well-being of adolescents, particularly among those from socioeconomically disadvantaged backgrounds.

## Data Availability

No datasets were generated or analysed during the current study.
